# Decreasing Blood Culture Collection in Hospitalized Patients with CAP, SSTI, and UTI

**DOI:** 10.1097/pq9.0000000000000705

**Published:** 2023-12-05

**Authors:** Monica D. Combs, Danica B. Liberman, Vivian Lee

**Affiliations:** From the * Division of Hospital Medicine, Children’s Hospital Los Angeles, Los Angeles, Calif.; † Department of Pediatrics, Keck School of Medicine of University of Southern California, Los Angeles, Calif.; ‡ Division of Emergency and Transport Medicine, Children’s Hospital Los Angeles, Los Angeles, Calif.; § Department of Population and Public Health Sciences, Keck School of Medicine, University of Southern California, Los Angeles, Calif.

## Abstract

**Background:**

Blood culture collection in pediatric patients with community-acquired pneumonia (CAP), skin and soft tissue infections (SSTI), and urinary tract infections (UTI) remains high despite evidence of its limited utility. We aimed to decrease the number of cultures collected in children hospitalized for CAP, SSTI, and UTI by 25% over 11 months.

**Methods:**

Quality improvement initiative at a children’s hospital among well-appearing patients aged 2 months or more to 18 years diagnosed with CAP, SSTI, or UTI. Our primary and secondary outcomes were blood culture collection rate and positivity rate, respectively. Interventions focused on three key drivers: academic detailing, physician awareness of personal performance, and data transparency.

**Results:**

Over the 2-year study period, there were 105 blood cultures collected in 223 hospitalized patients. Blood culture collection rates demonstrated special cause variation, decreasing from 63.5% to 24.5%. For patients with UTI, 86% (18/21) of blood cultures were negative, whereas 100% were negative for CAP and SSTI. All three patients with bacteremic UTI had a concurrent urine culture growing the same pathogen. Balancing measures remained unchanged, including escalation to a higher level of care and return to the emergency department or hospital within 14 days for the same infection.

**Conclusions:**

A multifaceted quality improvement approach can reduce blood culture collection for hospitalized patients with CAP, SSTI, and UTI without significant changes to balancing measures. Despite the reduction achieved, the near-universal negative culture results suggest continued overutilization and highlight the need for more targeted approaches to blood culture collection.

## INTRODUCTION

The growing costs of healthcare in the United States and a growing focus on reducing overuse highlight the importance of prioritizing our research efforts on diagnostic stewardship.^1,2^ Community-acquired pneumonia (CAP), skin and soft tissue infections (SSTI), and urinary tract infections (UTI) are among the top 10 diagnoses for pediatric hospital admissions, with CAP considered the second most costly diagnosis in the United States.^1,3^

Many recent studies conclude that obtaining blood cultures in well-appearing, healthy children with these diagnoses—CAP, SSTI, UTI—is of limited utility. In a nontoxic child with uncomplicated CAP, multiple studies have shown that routine blood cultures are unnecessary given the very low likelihood that a blood culture will be positive or result in a change in management.^4–8^ The low utility is partly driven by increased immunization against *Haemophilus influenzae type b* and *Streptococcus pneumoniae*, dramatically decreasing bacteremia rates.^9,10^ Among SSTI, despite the increasing prevalence of methicillin-resistant *Staphylococcus aureus*, evidence discourages routine collection of blood cultures in immunocompetent children.^11–14^ For both CAP and SSTI, multiple studies have identified a higher rate of contaminants than true pathogens.^4,5,7,12^ Finally, bacteremia infrequently occurs in children with UTI and, when identified, is nearly always identical to the urine culture isolate, signifying that blood cultures may represent a low-value test.^15,16^

Even when clinical management for CAP, SSTI, or UTI changes due to a positive blood culture, this change is often arbitrary and not evidence-based; thus, the benefit to the patient remains unclear.^4^ For instance, in infants with bacteremic UTI, outcomes are generally excellent despite a wide range in length of stay and duration of parenteral antibiotics.^17–19^ Additional studies on CAP found that increased resource utilization including blood culture collection, leads to longer length of stay, with no data to support improved benefit.^20,21^

Numerous studies, including several recent Pediatric Health Information System database studies, reveal high blood culture collection rates among these three diagnoses, with significant variability noted across hospitals.^8,20–22^ Data at our institution suggested similarly high collection rates and highlighted the need for local quality improvement (QI) efforts.

Our study aimed to decrease the blood culture collection rate in well-appearing children 2 months or more to 18 years old, hospitalized for CAP, SSTI, and UTI by 25% over an 11-month study period.

## METHODS

### Context

We conducted this QI initiative at a 413-bed urban academic freestanding children’s hospital. Patients aged 2 months or more to 18 years old admitted to the general wards service, both resident and nonresident teams, with a diagnosis of CAP, SSTI, or UTI were eligible for inclusion.

We collected data for this initiative concurrently with data collection for the Better Antibiotic Selection in Children (BASiC) study. BASiC is a multicenter Value in Inpatient Pediatrics Network collaborative QI project through the American Academy of Pediatrics. It is focused on improving antibiotic stewardship for CAP, SSTI, and UTI. In addition to the data collected for BASiC, we collected data on blood culture orders and results. We adopted our inclusion and exclusion criteria from the BASiC study criteria (Table 1). We defined the presence of a complex chronic condition (CCC) using the pediatric CCC classification system.^23^

**Table 1.** Inclusion and Exclusion Criteria

Inclusion criteria	Age >2 months to 18 years
	Admitted to hospital
	Discharge diagnosis ICD-10 codes for CAP, UTI, or SSTI*
	Prescribed antibiotics within first 24 hours of presentation
	Treated with full course of antibiotics for one of the above diagnoses
Exclusion criteria	Immunocompromised
	Ill-appearing
	Diagnosis of Cystic Fibrosis
	Patients with SSTI involving face, eyes, perineum; animal bites and postoperative wound infections
	Patients treated for >1 infection
	Anyone started on antibiotics for presumed infection first as outpatient or transferred from referring hospital

* ICD-10 codes: UTI: N39.0; N15.1; N30.0; N30.9; N10; CAP: J12.0, J12.1, J12.2, J12.89, J15.4, J15.0, J13, J18.1, J15.3, J15.8, J15.6, J15.211, J15.212, J16.8, A37.91, J18.0, J18.9, J11.00, J12.9; J15.9; SSTI: L03.xx, L02.xx; N61

The baseline period spanned 13 months, from April 2020 through April 2021; we implemented interventions starting in May 2021 and collected data through March 2022. For each month of the 2-year study period, we included a random sample of up to 20 unique patients per month. We excluded patients transferred from a referring facility. The breakdown of eligible patients by diagnosis varied month-to-month based on census and the number of patients meeting eligibility criteria.

### Planning the Interventions

Project leaders included a pediatric hospital medicine fellow, a pediatric hospitalist, and a pediatric emergency medicine (PEM) attending. Key stakeholders included pediatric hospitalists, PEM physicians, pediatric residents, and emergency department (ED) nurses. The project leaders met regularly to discuss tests of change and review stakeholder feedback.

**Fig. 1.**
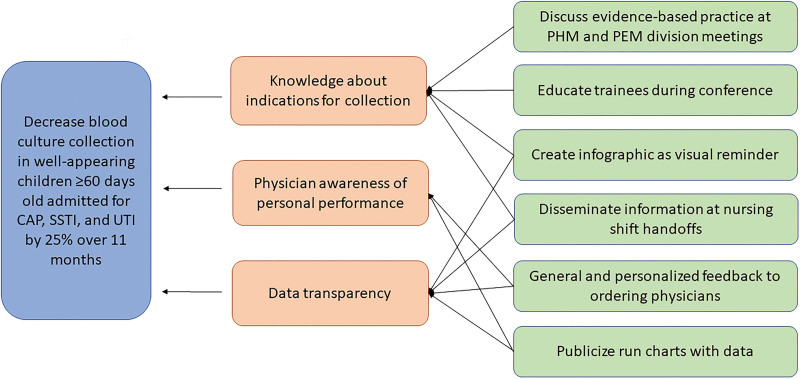
Key driver diagram. PEM, pediatric emergency medicine; PHM, pediatric hospital medicine.

We solicited initial perspectives on blood culture collection informally via in-person discussions and email responses from attending physicians, hospital medicine fellows, and pediatric residents. Through this process, we identified key barriers to decreasing our collection rates, including lack of provider knowledge of relevant evidence-based literature, lack of feedback to ordering physicians on blood culture results, and current hospital culture reinforcing blood culture ordering. We also felt that ED nursing played a role in blood culture collection; factors included nursing prompting PEM physicians to collect blood cultures on initial assessment to limit the number of blood draws. Based on these reported barriers from stakeholders, we identified potential interventions to help reduce and remove these barriers. Our team used this information to develop a fishbone diagram (See **figure 1, Supplemental Digital content 1,** which shows PEM and PHM. http://links.lww.com/PQ9/A527) and key driver diagram (Fig. 1) to guide the implementation of sequential plan-do-study-act (PDSA) cycles.

### QI Activities

Interventions focused on three key drivers for decreasing blood culture collection rates: (1) academic detailing, (2) physician awareness of personal performance, and (3) data transparency. We implemented changes through a series of PDSA cycles and revisited the initial key driver diagram to address areas of continued need. In addition, throughout and after each PDSA cycle, we gathered feedback from our key stakeholders verbally and electronically to help guide iterative improvements.

#### Academic detailing:

At the start of the intervention period, we led interactive academic detailing sessions^24^ with the divisions of hospital medicine and emergency medicine and pediatric residents. These sessions highlighted evidence-based care and our key messages while allowing us to address any barriers and/or objections. We distributed the presentation slides electronically to the head of ED nursing for dissemination to the ED nursing staff for asynchronous education and discussions. To account for new interns beginning during the project intervention, we led regular refresher sessions with residents throughout the study period.

#### Physician awareness of personal performance:

We performed additional manual chart reviews on all patients with a blood culture collected to identify the ordering physicians and review the documented reasoning for blood culture ordering. Every 2 months, we sent individualized emails to physicians who had ordered blood cultures, sharing a brief reminder of the patient presentation and the project goals and progress to date.

#### Data transparency:

We encouraged practice change by describing colleagues’ current blood culture ordering behavior and emphasizing the significant proportion of negative blood culture results. At 2- to 3-month intervals, we shared updated run charts with the entire division of emergency medicine and all pediatric residents showing the proportion of blood cultures per hospitalized patient with one of the three diagnoses collected per month and the rate of negative blood culture results.

### Measures

The primary outcome measure was the percentage of patients hospitalized with CAP, UTI, or SSTI who had a blood culture collected at any point during their hospitalization. The secondary outcome metric was the positive blood culture rate compared with negative and contaminant blood culture rates. Balancing measures included escalation to a higher level of care (ie, transfer to the pediatric intensive care unit) and return to the ED or hospital within 14 days of discharge for the same infection. We obtained data via manual chart review and entered the data into REDCap.^25^ A small group of physicians from the divisions of hospital medicine, emergency medicine, infectious disease, and pediatric residency who were part of the larger parent BASiC study and had received standardized training in the data collection and entry for that study abstracted data from the electronic medical record. In addition, to improve data accuracy and monitor data collection and entry, a hospital medicine attending reviewed all submitted patients to confirm they met inclusion criteria. A hospital medicine fellow manually reviewed all charts of patients with blood cultures ordered.

### Analysis

We used a statistical process control p-chart (QI Macros, SPC Software) to analyze the blood culture collection rate and track our balancing measures. We calculated the collection rate as total blood cultures divided by the total number of included patients, which varied monthly based on how many patients met our eligibility criteria. We applied established rules for determining special cause variation to shift the centerline down after the identified breakpoint.^26^

### Ethical Considerations

This study was not human subject research. Our hospital’s institutional review board provided a QI exemption.

## RESULTS

**Fig. 2.**
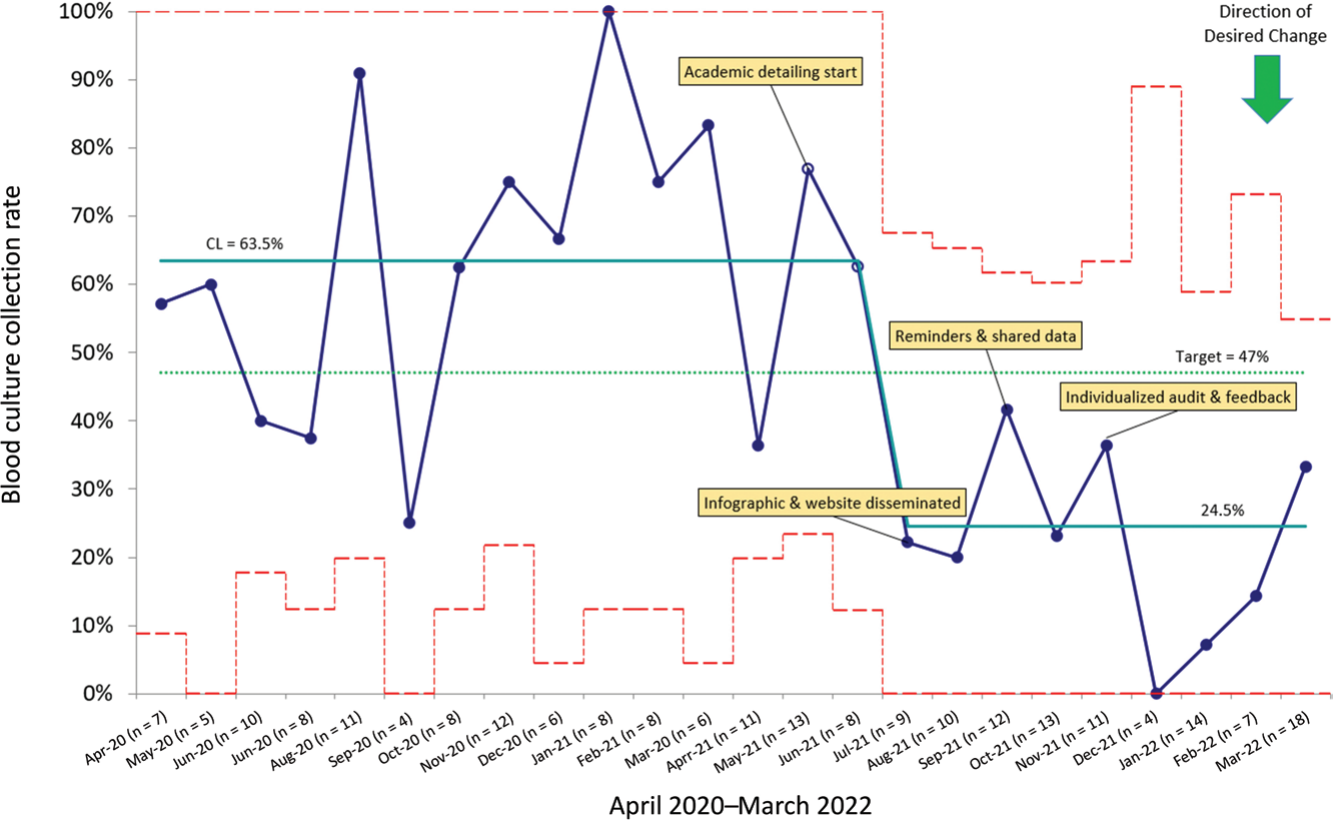
Blood cultures collected in hospitalized patients with CAP, SSTI, and UTI.

Of the 223 patients who met inclusion criteria throughout the 2-year study period, 104 were in the baseline period and 119 in the intervention period. The blood culture collection rate was 63.5% (66/104) during the baseline period from April 2020 to April 2021. In July 2021, we observed a desirable downward shift meeting criteria for a special cause, resulting in a 24.5% (24/98) collection rate sustained until our study period’s end (Fig. 2). As mentioned above, we included 104 patients for analysis during the baseline period. The proportion of blood cultures collected by diagnosis during the baseline period was 46.7% (7/15) for CAP, 69.7% (23/33) for SSTI, and 64.3% (36/56) for UTI. The postintervention period included 119 patients. The proportion of blood cultures collected by diagnosis postintervention was 9.7% (4/41) for CAP, 41.2% (14/34) for SSTI, and 47.7% (21/44) for UTI.

The average age of our cohort was 5.1 years old. Most patients were female, Hispanic, had government insurance, and did not have a CCC. Each encounter represents a discrete patient; we did not include any patients more than once.

### Blood Culture Results

Ninety-six percent of total cultures (101/105) were negative throughout the baseline and intervention periods. One culture was treated as a contaminant (coagulase-negative Staphylococcus). The three remaining positive cultures were all in aged 3 months or older infants with *E. coli* bacteremia with concurrent UTIs growing the same pathogen on urine culture. For all patients with CAP and SSTI, 100% of blood cultures were negative.

### Balance Measures

There were no significant changes in our balance measures during the study. During the baseline period, four patients returned to the ED or hospital within 14 days of discharge for the same infection, and none escalated to a higher level of care. During the intervention period, four patients returned to the ED within 14 days of discharge for the same infection, and no patients escalated to a higher level of care. None of these patients had identified bacteremia.

## DISCUSSION

Our study reveals that QI methodology can effectively decrease blood culture collection rates. Specifically, our study resulted in a 61% decrease in provider ordering behavior, which surpassed our goal. Our data reinforce existing literature that most blood cultures in nontoxic patients hospitalized with CAP, SSTI, or UTI are negative. Therefore, their routine collection is unnecessary. Our success in targeting blood culture overutilization is an important step toward mitigating low-value care and the potential risks for harm.

This project was unique in focusing on decreasing blood culture utilization across multiple diagnoses. Specific efforts of our project that seemed most helpful include creating a multi-disciplinary team, compiling relevant literature and disseminating the information via interactive sessions, making an infographic (**See figure 2, Supplemental Digital Content 2,** which shows FGC and MRSA. http://links.lww.com/PQ9/A528) with high visibility and high yield concise take-home points, and providing audit and feedback to ordering physicians. Notably, we achieved special cause variation after the initial interventions of academic detailing and dissemination of the infographic. With this project, we initiated the first steps in a shared mental model for decreasing unnecessary blood cultures but changing the hospital culture is a long-term improvement step that takes time to integrate fully. Literature suggests that it takes 17 years to translate evidence to clinical practice, and deimplementation research highlights that it is even harder to take away something already ingrained in our usual culture.^27,28^ Qualitative research identifies that deimplementation requires unique strategies to mitigate the psychocognitive challenges of doing less.^29^ By widely sharing evidence and implementing this local project within the context of a national collaborative, we believe this helps normalize the behavior, permits physicians to change practices, and contributes to a growing institutional culture of high-value care.

Patients admitted with CAP primarily drove the improvement in the blood culture collection rate, with SSTI also playing a significant but smaller role. Though we did not design our study to determine why one diagnosis showed more improvement than the others, one hypothesis for CAP is that the likelihood of obtaining a blood culture for CAP was already lower at baseline; if ordering blood cultures is already less routine for physicians, it may be easier to change behavior to order even fewer blood cultures. Another possible contributing factor is that the number of CAP patients in the baseline group was less than the other diagnoses; therefore, the baseline collection rate may have been proportionally more elevated when compared with the other diagnoses. For SSTI, based on additional chart review, more significant improvement may have been thwarted by physicians ruling out the diagnosis of osteomyelitis, a diagnosis for which blood cultures have a higher yield and may be indicated. We noted smaller absolute improvements in blood culture collection rates in UTI. For patients with UTI, symptoms can be more undifferentiated compared with CAP or SSTI and thus, may have compelled physicians to initiate a more expanded work-up. Additionally, our study included patients 60 days old or more; however, many physicians still consider less than 90 days old as the cutoff for pursuing a febrile infant work-up with both blood and urine cultures. Finally, our results must be qualified in the context of the COVID-19 pandemic, in a time of elevated concern for multisystem inflammatory syndrome in children, and consequently, a period when a work-up for pediatric fever was more expansive.^30^ This suggests that the results of similar QI efforts to reduce blood culture collection during a nonpandemic era could be even more pronounced.

A few prior QI efforts in pediatric patients have also demonstrated a successful reduction in blood culture utilization without significant changes to balancing measures.^31,32^ Lee et al reduced unnecessary testing, including blood cultures, by 35% in uncomplicated SSTIs by identifying the appropriate patient population to limit testing, addressing the current culture, and using the electronic medical record to guide clinical decision-making.^33^ Another initiative by Rogers et al. successfully decreased blood culture collection rates in CAP from 24% to 14% through interventions focusing on guideline implementation, data sharing, and provider education.^34^ In the pediatric intensive care setting, Woods-Hill et al targeted diagnostic stewardship to reduce blood culture collection safely, and secondarily antibiotic use, by 33% among their multicenter collaborative.^35^

Our study has several important limitations. We collected our data with a larger study involving multiple providers in the chart review process. To confirm data extraction consistency and accuracy, this study author manually re-reviewed patients with a collected blood culture. Our study also spanned the COVID-19 pandemic; so provider ordering behavior may have differed in this context. To help mitigate this, we adjusted our baseline period to show collection rate patterns during the pandemic. This project used retrospective data and relied on discharge diagnoses rather than admission diagnoses, which may have narrowed our study population and affected the overall collection rates observed. However, this would not affect the integrity of the significant improvement noted over time, as eligibility criteria were consistent throughout the study. In this project, we addressed multiple diagnoses concurrently, which may have limited the improvement we could have seen if the focus had been solely on one diagnosis. Our relatively small sample size precluded meaningful subgroup analysis by diagnosis, which may have affected the pooled results observed. Finally, because this QI project occurred at a single site, our data and improvement efforts may not be generalizable. However, our overarching key drivers may be effective starting points for other institutions looking to reduce unnecessary blood culture collection.

## CONCLUSIONS

We demonstrated that a multifaceted approach focused on academic detailing, increasing physician awareness of personal performance, and data transparency can decrease blood culture use for hospitalized pediatric patients with CAP, SSTI, and UTI. However, the persistently high collection rates and negative culture results for patients with these diagnoses suggest that there is room for improvement in addressing overutilization and a need for clearer guidelines and guidance on when to collect blood cultures. Focus areas for future studies include broader QI efforts to decrease blood culture overutilization and rigorous research to identify better which subset of patients may benefit from the identification of bacteremia.

## Supplementary Material



## References

[1] Kaiser SV Rodean J Coon ER. Common diagnoses and costs in pediatric hospitalization in the US. JAMA Pediatr. 2022;176:316–318. 34962555 10.1001/jamapediatrics.2021.5171 PMC8715384

[2] Gill PJ Anwar MR Thavam T; Pediatric Research in Inpatient Setting (PRIS) Network. Identifying conditions with high prevalence, cost, and variation in cost in US Children’s hospitals. JAMA Netw Open. 2021;4:e2117816. 34309667 10.1001/jamanetworkopen.2021.17816 PMC8314139

[3] Witt WP Weiss AJ Elixhauser A. Overview of hospital stays for children in the United States, 2012: statistical brief #187. Healthcare Cost and Utilization Project (HCUP) Statistical Briefs; 2006. 25695124

[4] Parikh K Davis AB Pavuluri P. Do we need this blood culture? Hosp Pediatr. 2014;4:78–84. 24584976 10.1542/hpeds.2013-0053

[5] Neuman MI Hall M Lipsett SC; Pediatric Research in Inpatient Settings Network. Utility of blood culture among children hospitalized with community-acquired pneumonia. Pediatrics. 2017;140:e20171013. 28835382 10.1542/peds.2017-1013 PMC5574722

[6] Lipsett SC Hall M Ambroggio L. Predictors of bacteremia in children hospitalized with community-acquired pneumonia. Hosp Pediatr. 2019;9:770–778. 31519736 10.1542/hpeds.2019-0149

[7] Heine D Cochran C Moore M. The prevalence of bacteremia in pediatric patients with community-acquired pneumonia: guidelines to reduce the frequency of obtaining blood cultures. Hosp Pediatr. 2013;3:92–96. 24340408 10.1542/hpeds.2012-0050

[8] House SA Hall M Ralston SL. Development and use of a calculator to measure pediatric low-value care delivered in US children’s hospitals. JAMA Netw Open. 2021;4:e2135184. 34967884 10.1001/jamanetworkopen.2021.35184 PMC8719236

[9] Stoll ML Rubin LG. Incidence of occult bacteremia among highly febrile young children in the era of the pneumococcal conjugate vaccine: a study from a children’s hospital emergency department and urgent care center. Arch Pediatr Adolesc Med. 2004;158:671–675. 15237067 10.1001/archpedi.158.7.671

[10] Johnson DP Lee V Gourishankar A. Things we do for no reason: routine blood culture acquisition for children hospitalized with community-acquired pneumonia. J Hosp Med. 2020;15:107–110. 31532737 10.12788/jhm.3279

[11] Wathen D Halloran DR. Blood culture associations in children with a diagnosis of cellulitis in the era of methicillin-resistant *Staphylococcus aureus*. Hosp Pediatr. 2013;3:103–107. 24340410 10.1542/hpeds.2012-0059 PMC3998118

[12] Malone JR Durica SR Thompson DM. Blood cultures in the evaluation of uncomplicated skin and soft tissue infections. Pediatrics. 2013;132:454–459. 23918896 10.1542/peds.2013-1384

[13] Zwemer E Stephens JR. Things we do for no reason: blood cultures for uncomplicated skin and soft tissue infections in children. J Hosp Med. 2018;13:496–499. 29964272 10.12788/jhm.2984

[14] Stevens DL Bisno AL Chambers HF; Infectious Diseases Society of America. Practice guidelines for the diagnosis and management of skin and soft tissue infections: 2014 update by the Infectious Diseases Society of America. Clin Infect Dis. 2014;59:e10–e52. 24973422 10.1093/cid/ciu444

[15] Newman TB Bernzweig JA Takayama JI. Urine testing and urinary tract infections in febrile infants seen in office settings: the pediatric research in office settings’ febrile infant study. Arch Pediatr Adolesc Med. 2002;156:44–54. 11772190 10.1001/archpedi.156.1.44

[16] Pitetti RD Choi S. Utility of blood cultures in febrile children with UTI. Am J Emerg Med. 2002;20:271–274. 12098170 10.1053/ajem.2002.33786

[17] Roman HK Chang PW Schroeder AR. Diagnosis and management of bacteremic urinary tract infection in infants. Hosp Pediatr. 2015;5:1–8. 25554753 10.1542/hpeds.2014-0051

[18] Olson J Franz-O’Neal E Cipriano FA. Impact of early oral antibiotic therapy in infants with bacteremic urinary tract infections. Hosp Pediatr. 2022;12:632–638. 35726551 10.1542/hpeds.2021-006479

[19] Nama N Donken R Pawliuk C; INSIGHTSCOPE TEAM. Treatment of UTIs in infants <2 months: a living systematic review. Hosp Pediatr. 2021;11:1017–1030. 34446534 10.1542/hpeds.2021-005877

[20] Brogan TV Hall M Williams DJ. Variability in processes of care and outcomes among children hospitalized with community-acquired pneumonia. Pediatr Infect Dis J. 2012;31:1036–1041. 22653486 10.1097/INF.0b013e31825f2b10 PMC3504613

[21] Leyenaar JK Lagu T Shieh MS. Variation in resource utilization for the management of uncomplicated community-acquired pneumonia across community and children’s hospitals. J Pediatr. 2014;165:585–591. 24973795 10.1016/j.jpeds.2014.04.062 PMC4158451

[22] Stephens JR Hall M Markham JL. Variation in proportion of blood cultures obtained for children with skin and soft tissue infections. Hosp Pediatr. 2020;10:331–337. 32184289 10.1542/hpeds.2019-0317

[23] Feudtner C Feinstein JA Zhong W. Pediatric complex chronic conditions classification system version 2: updated for ICD-10 and complex medical technology dependence and transplantation. BMC Pediatr. 2014;14:199. 25102958 10.1186/1471-2431-14-199 PMC4134331

[24] Knox L Brach C Fischer M. Primary Care Practice Facilitation Curriculum (Module 16). AHRQ Publication No 15-0060-EF. Agency for Healthcare Research and Quality; September 2015.

[25] Harris PA Taylor R Thielke R. Research electronic data capture (REDCap)—a metadata-driven methodology and workflow process for providing translational research informatics support. J Biomed Inform. 2009;42:377–381. 18929686 10.1016/j.jbi.2008.08.010 PMC2700030

[26] Benneyan JC Lloyd RC Plsek PE. Statistical process control as a tool for research and healthcare improvement. Qual Saf Health Care. 2003;12:458–464. 14645763 10.1136/qhc.12.6.458 PMC1758030

[27] Morris ZS Wooding S Grant J. The answer is 17 years, what is the question: understanding time lags in translational research. J R Soc Med. 2011;104:510–520. 22179294 10.1258/jrsm.2011.110180 PMC3241518

[28] Kulkarni SA Leykum LK Moriates C. Deimplementation: discontinuing low-value, potentially harmful hospital care. J Hosp Med. 2021;16:63. 33357335 10.12788/jhm.3563 PMC7768917

[29] McDaniel CE House SA Ralston SL. Behavioral and psychological aspects of the physician experience with deimplementation. Pediatr Qual Saf. 2022;7:e524. 35071960 10.1097/pq9.0000000000000524 PMC8782108

[30] Liang CS Forster C Williams AE. High-value care during the COVID-19 pandemic: lessons learned and future opportunities. Hosp Pediatr. 2022;12:e216–e218. 35641475 10.1542/hpeds.2021-006511

[31] Lemberg KM Koontz DW Young DJ. Trainee-led engagement of the care team improves application of an institutional blood culture clinical decision algorithm to pediatric oncology inpatients: a single-institution quality improvement project. Pediatr Qual Saf. 2022;7:e545. 35369412 10.1097/pq9.0000000000000545 PMC8970086

[32] Woods-Hill CZ Lee L Xie A. Dissemination of a novel framework to improve blood culture use in pediatric critical care. Pediatr Qual Saf. 2018;3:e112. 30584639 10.1097/pq9.0000000000000112 PMC6221585

[33] Lee B Hershey D Patel A. Reducing unnecessary testing in uncomplicated skin and soft tissue infections: a quality improvement approach. Hosp Pediatr. 2020;10:129–137. 31941651 10.1542/hpeds.2019-0179

[34] Rogers AJ Lye PS Ciener DA. Using quality improvement to change testing practices for community-acquired pneumonia. Pediatr Qual Saf. 2018;3:e105. 30584632 10.1097/pq9.0000000000000105 PMC6221590

[35] Woods-Hill CZ Colantuoni EA Koontz DW; Bright STAR Authorship Group. Association of diagnostic stewardship for blood cultures in critically Ill children with culture rates, antibiotic use, and patient outcomes: results of the bright STAR collaborative. JAMA pediatrics 2022;176:690–698. 35499841 10.1001/jamapediatrics.2022.1024 PMC9062771

