# Supplementary material for: Decreasing Blood Culture Collection in Hospitalized Patients with CAP, SSTI, and UTI
Source: Pediatr Qual Saf. 2023 Dec 5;8(6):e705. doi: 10.1097/pq9.0000000000000705 (PMC10697617; doi:10.1097/pq9.0000000000000705)
Supplement: Supplementary file 1 [file pqs-8-e705-s001.pdf]

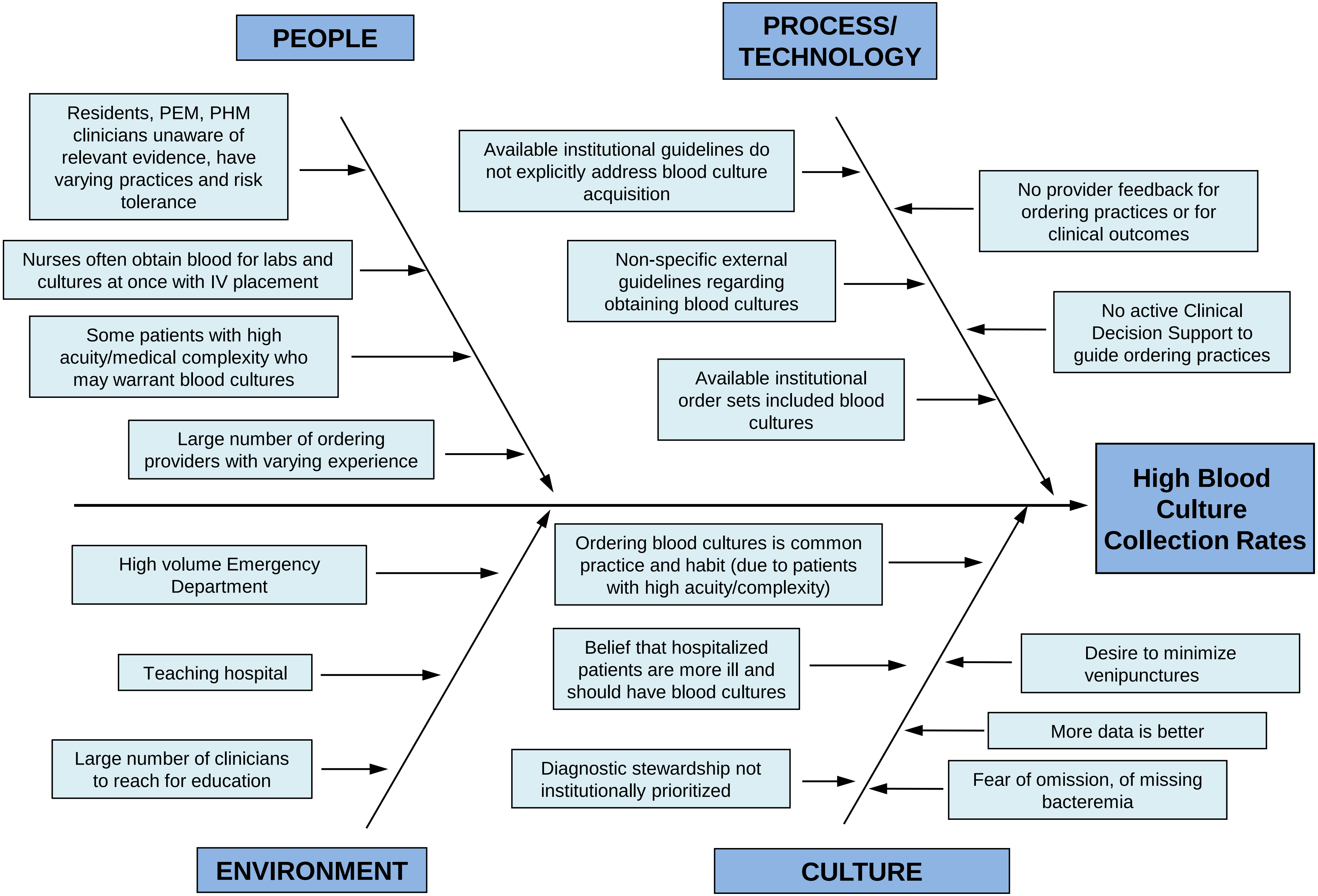
PEOPLE
PROCESS/ TECHNOLOGY
Residents, PEM, PHM clinicians unaware of relevant evidence, have varying practices and risk tolerance
Nurses often obtain blood for labs and cultures at once with IV placement
Some patients with high acuity/medical complexity who may warrant blood cultures
Large number of ordering providers with varying experience
Available institutional guidelines do not explicitly address blood culture acquisition
Non-specific external guidelines regarding obtaining blood cultures
Available institutional order sets included blood cultures
No provider feedback for ordering practices or for clinical outcomes
No active Clinical Decision Support to guide ordering practices
High Blood Culture Collection Rates
High volume Emergency Department
Teaching hospital
Large number of clinicians to reach for education
Ordering blood cultures is common practice and habit (due to patients with high acuity/complexity)
Belief that hospitalized patients are more ill and should have blood cultures
Diagnostic stewardship not institutionally prioritized
Desire to minimize venipunctures
More data is better
Fear of omission, of missing bacteremia
ENVIRONMENT
CULTURE
