# Supplementary material for: Decreasing Blood Culture Collection in Hospitalized Patients with CAP, SSTI, and UTI
Source: Pediatr Qual Saf. 2023 Dec 5;8(6):e705. doi: 10.1097/pq9.0000000000000705 (PMC10697617; doi:10.1097/pq9.0000000000000705)
Supplement: Supplementary file 2 [file pqs-8-e705-s002.pdf]

BASiC: Better Antibiotic Selection in Children

An AAP Value in Inpatient Pediatrics (VIP) Multicenter QI Collaborative

CHLA Emergency Department, Infectious Diseases/Antibiotic Stewardship, Pediatric Residency, General Pediatrics, Hospital Medicine

The BASiCs of Antibiotics — Be Timely, Targeted, TEMPORARY

BE ANTIBIOTICS AWARE — SMART USE, BEST CARE — https://www.cdc.gov/antibiotic-use

For **non-toxic, immunocompetent** patients seen in the ED or hospitalized with **uncomplicated CAP, SSTI,** and **UTI,** please consider:

CAP

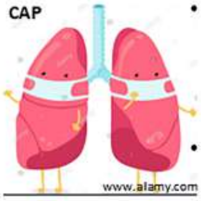

- Narrow spectrum therapy with **high dose amoxicillin or ampicillin** (clinda if allergic)
- Limit routine azithromycin: no clear benefit as combo treatment; not good for *S. pneumo* coverage due to resistance

SSTI

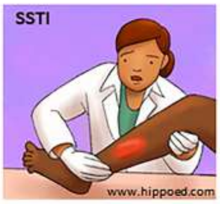

- **First generation cephalosporins** (FGC - cephalexin, cefazolin) for patients with NON-PURULENT SSTI and LOW RISK MRSA
- Purulent: **FGC** may be ok; **clindamycin** if suspect MRSA

UTI

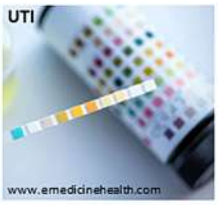

- **Cephalexin** when patients can tolerate oral antibiotics
- Discontinuing antibiotics if they do not meet AAP diagnostic criteria for UTI

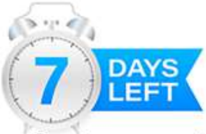

- Shorter courses of total 7 days is sufficient in most cases of **simple** CAP, SSTI, UTI.
  Yes, even pyelonephritis!
- Each day of antibiotics can add risk for adverse effects and harms to our patients – **so every dose counts**!

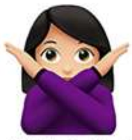

Think twice before obtaining blood cultures – very low yield in a result that will change your management:

- Low rates of true positivity
- Lower rates of an organism resistant to your empiric treatment
- Even with true secondary bacteremia, does not necessarily change duration of treatment

Download the VIP BASiC Decision Aid app!

Don't forget to use our **MAPs** to help you with your treatment!

Find links to evidence + other guidelines here!
